# Subjective results of joint lavage and viscosupplementation in hemophilic arthropathy

**DOI:** 10.1590/1413-785220152302145885

**Published:** 2015

**Authors:** Márcia Uchoa de Rezende, Thiago Bittencourt Carvalho Rosa, Thiago Pasqualin, Renato Frucchi, Erica Okazaki, Paula Ribeiro Villaça

**Affiliations:** 1 Department of Orthopedics and Traumatology, Hospital das Clinicas da Faculdade de Medicina da Universidade de São Paulo, São Paulo, SP, Brazil; 2 Hematology Service, Hospital das Clínicas da Faculdade de Medicina da Universidade de São Paulo, São Paulo, SP, Brazil

**Keywords:** Hemophilia, Osteoarthritis, knee, Hyaluronic acid/administration & dosage, Hyaluronic acid/therapeutic use

## Abstract

**OBJECTIVE:**

To assess whether joint lavage, viscosupplementation and triamcinolone improve joint pain, function and quality of life in patients with severe hemophilic arthropathy.

**METHODS:**

Fourteen patients with knee and/or ankle hemophilic arthritis with and without involvement of other joints underwent joint lavage and subsequent injection of hylan G-F20 and triamcinolone in all affected joints. The patients answered algo-functional questionnaires (Lequesne and WOMAC), visual analog scale for pain (VAS) and SF-36 preoperatively, and at one, three, six and twelve months postoperatively.

**RESULTS:**

Sixteen knees, 15 ankles, 8 elbows and one shoulder were treated in 14 patients. Six patients had musculoskeletal bleeding [ankle (1), leg muscle (2) and knees (4)] at 3 months affecting the results. Pain did not improve significantly. Function improved (WOMAC p=0.02 and Lequesne p=0.01). The physical component of SF-36 improved at all time points except at 3 months, with best results at one-year follow-up (baseline = 33.4; 1 month = 39.6; 3 months= 37.6; 6 months 39.6 and 1 year = 44.6; p < 0.001).

**CONCLUSION:**

Joint lavage followed by injection of triamcinolone and hylan G-F20 improves function and quality of life progressively up to a year, even in severe hemophilic arthropathy. *Level of Evidence IV, Case Series.*

## INTRODUCTION

Young hemophiliac patients sequelaed by multiple joint bleeds, awaiting surgical treatment can improve function and quality of life of multiple joints by viscossuplementation^1^^,^ 2 allowing proper rehabilitation and the correct choice of surgical procedure of each joint and their order. The viscosupplementation outcomes can be improved by joint washing and adding of triamcinolone.^2^^-^ 6

The hylan G-F20 is the only market drug with proven application in a single dose (6 mL) that shows results equal to or superior to the injection of 2 mL por week for three weeks in the knee,^7^^,^ 8 allowing a single treatment in patients who must receive clotting factor before the procedure, a factor of higher cost than the cost of viscosupplementation itself.

The objective of this study is to evaluate the effectiveness of the treatment, consisting of joint lavage followed by infiltration with corticosteroids and hylan G-F 20 regarding pain relief and improved function and quality of life over a year.

## METHODS

This case series was conducted at the Osteometabolic Diseases Group, Department of Orthopedics and Traumatology, *Hospital das Clínicas, Universidade de São Paulo* (IOT-HC-USP) with the approval of the Ethics Committee for Research Project Analysis (CAPPesq) number 8847/12.

In order to participate in the study patients should be severe hemophiliacs type A or B, from the hemophilia outpatient at the Department of Hematology, HC-FMUSP, with one or more joints with chronic arthropathy (Arnold and Hilgartner grades III or IV)^9^ or stage of joint destriction (grade V) having the knee and/or the ankle as at least one of the impaired articulations; presenting no allergies to substances; no previous intra-articular infiltration or surgery in target joints in the last three months before the procedure; and able to understand and agree with the Informed Consent.

Among the exclusion criteria there would be abandoning follow up; new joint infiltration during the study period; having a severe adverse reaction to the procedure or having infection in the target articulation during the study period.

**Figure 1.**
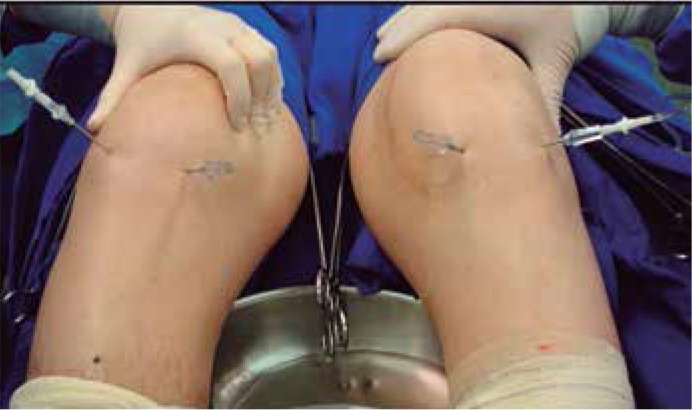
Arthroscopic lavage of left and right knees.

**Figure 2.**
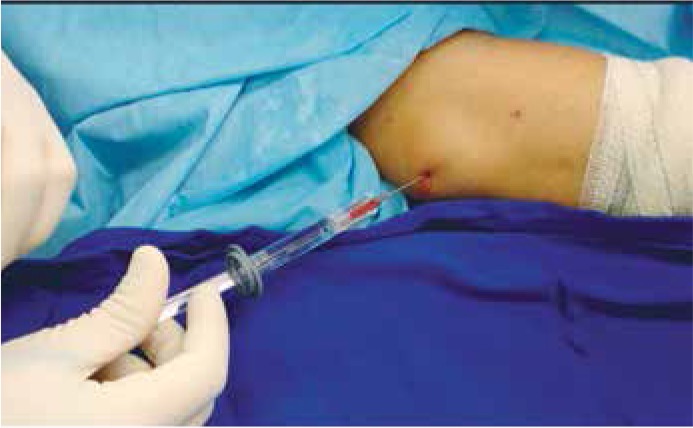
Infiltration with Hylan G-F20 (2 mL) after elbow lavage.

The procedure was performed under anesthesia in the operating room under asepsis and surgical scrub as an outpatient. The clotting factor replacement for the procedure was accompanied by hematologists. The knee joint was punctured by two Jelcos 14 (inlet and outlet) connected to a physiological serum catheter and a surgical aspirator, respectively. (Figure 1) The joint was washed out until a translucent liquid was obtained, with non-bleeding aspect. The joints of the elbow and ankle were punctured with two needles (40x12 or Jelco 14, Figure 2). The infusion (washing out) and aspiration of saline solution were carried out by a syringe until a translucent liquid was obtained. After emptying the joint, it was infiltrated with one ampule of hyaluronic acid (Synvisc^(r)^ One^TM)^ with 1 mL of triamcinolone diluted in 5 mL of ropivacaine in the knee joints and one ampule (2 mL) of hyaluronic acid (Synvisc^(r))^ with 1 mL of triamcinolone diluted in 2 mL of ropivacaine in the ankles, elbows and shoulder. Patients were discharged on the same day, with compressive bandaging and guidance of relative rest for two to three days. 

All patients responded subjective questionnaires regarding their overall quality of life and about arthropathy. We used the SF-36 (Short Form Health Survey - 36),^10^ for overall quality of life, WOMAC (Western Ontario and McMaster Universities Osteoarthritis Index^11 ^and Lequesne^12^ on the joint function and arthropaty. These questionnaires were answered the day before the procedure and after one, three, six and twelve months. Pain was assessed by the Visual Analog Scale (VAS)^13^ and the WOMAC pain component (WOMAC pain).^11^

All patients were followed by the hematology team for adequate control of the clotting disorder. 

### Statistical Analysis

Functionality and quality of life scales were described according to time points with mean and standard deviation and intervals with 95% confidence for the average.

The assessed parameters were compared according to time points with generalized estimating equations with symmetric component correlation matrix between times, with normal marginal distribution and identity link function, followed by Bonferroni multiple comparisons between times when statistically significant differences to identify at what time there was a change in the parameters.

The results were illustrated using graphs of mean profiles with their standard errors and the tests were performed with a significance level of 5%.

## RESULTS

Sixteen knees, 15 ankles, eight elbows and one shoulder were treated in 14 patients with a mean age of 23.7 years old, ranging from 13 to 37 years old. Six patients had musculoskeletal bleeding (one ankle, four knees and two leg muscle bleeding) close to the three months evaluation, affecting the results.

Table 1 shows that pain (VAS and WOMAC pain) did not improve significantly, but the function given by WOMAC, Lequesne and the physical component of quality of life showed significant mean change between time points in hemophilia patients (p <0.05).

**Table 1.** Description of functionality and quality of life questionnaires according to momentos and results of the comparative tests between times.

Variável	Time	Mean	St. Dev	N	CI (95%)		p
					Inferior	Supeior	
WOMAC	Pre	32.4	22.0	14	20.9	43.9	0.023
	1 month	24.1	19.1	14	14.2	34.1	
	3 months	25.3	18.0	13	15.5	35.1	
	6 months	23.5	18.0	14	14.1	32.9	
	1 year	22.4	19.0	13	12.1	32.7	
WDOR	Pre	6.3	4.0	14	4.2	8.4	0.064
	1 month	4.3	3.7	14	2.3	6.2	
	3 months	5.9	4.6	13	3.4	8.4	
	6 months	5.4	4.3	14	3.1	7.6	
	1 year	4.6	3.7	13	2.6	6.6	
EVA	Pre	45.7	27.4	14	31.4	60.0	0.684
	1 month	35.6	23.3	14	23.4	47.8	
	3 months	42.0	24.6	13	28.6	55.4	
	6 months	42.3	21.2	14	31.2	53.4	
	1 year	38.2	22.3	13	26.0	50.3	
Lequesne	Pre	10.1	3.9	14	8.1	12.2	0.011
	1 month	7.3	3.9	14	5.3	9.4	
	3 months	7.7	4.5	13	5.3	10.1	
	6 months	8.8	4.4	14	6.5	11.0	
	1 year	7.9	4.5	13	5.4	10.3	
PCS	Pre	33.4	7.4	14	29.5	37.2	<0.001
	1 month	39.6	7.9	14	35.4	43.7	
	3 months	37.6	9.9	13	32.2	43.0	
	6 months	39.6	8.7	14	35.0	44.1	
	1 year	44.6	10.2	13	39.1	50.2	
MCS	Pre	50.6	6.9	14	47.0	54.2	0.772
	1 month	52.5	8.5	14	48.0	56.9	
	3 months	53.0	10.8	13	47.2	58.9	
	6 months	51.9	7.7	14	47.8	55.9	
	1 year	52.2	7.4	13	48.2	56.2	

EEG Results.

In Table 2, we can see the improvement in the WOMAC function score which was reduced from pre-treatment to one year follow-up (p=0.035), Lequesne decreased in average from pre-treatment to one month and three month follow-up (p=0.014 and p=0.044 respectively) and the quality of life score in the physical component increased in average from pre-treatment to one month, six months and one year follow-up (p <0.05).

**Table 2.** Results of multiple comparisons between times for functionality and quality of life questionnaires that showed differences between time points.

Variable	Comparisons		Mean Difference	Standard Error	gl	p	CI (95%)	
							Inferior	Superior
WOMAC	Pre	1 month	8.29	3.34	1	0.130	-1.08	17.65
	Pre	3 months	7.94	3.41	1	0.200	-1.64	17.52
	Pre	6 months	8.93	3.34	1	0.074	-0.43	18.29
	Pre	1 year	9.98	3.41	1	0.035	0.40	19.56
	1 month	3 months	-0.34	3.41	1	>0.999	-9.93	9.24
	1 month	6 months	0.64	3.34	1	>0.999	-8.72	10.01
	1 month	1 year	1.70	3.41	1	>0.999	-7.89	11.28
	3 months	6 months	0.99	3.41	1	>0.999	-8.60	10.57
	3 months	1 year	2.04	3.49	1	>0.999	-7.76	11.84
	6 months	1 year	1.05	3.41	1	>0.999	-8.53	10.64
Lequesne	Pre	1 month	2.82	0.88	1	0.014	0.35	5.29
	Pre	3 months	2.57	0.90	1	0.044	0.04	5.10
	Pre	6 months	1.39	0.88	1	>0.999	-1.08	3.87
	Pre	1 year	2.10	0.90	1	0.199	-0.43	4.63
	1 month	3 months	-0.25	0.90	1	>0.999	-2.78	2.28
	1 month	6 months	-1.43	0.88	1	>0.999	-3.90	1.04
	1 month	1 year	-0.72	0.90	1	>0.999	-3.25	1.81
	3 months	6 months	-1.18	0.90	1	>0.999	-3.71	1.36
	3 months	1 year	-0.47	0.92	1	>0.999	-3.06	2.12
	6 months	1 year	0.71	0.90	1	>0.999	-1.83	3.24
PCS	Pre	1 month	-6.20	2.18	1	0.044	-12.32	-0.08
	Pre	3 months	-5.05	2.23	1	0.236	-11.30	1.21
	Pre	6 months	-6.20	2.18	1	0.044	-12.32	-0.08
	Pre	1 year	-10.80	2.23	1	<0.001	-17.06	-4.54
	1 month	3 months	1.15	2.23	1	>0.999	-5.10	7.41
	1 month	6 months	0.00	2.18	1	>0.999	-6.12	6.12
	1 month	1 year	-4.60	2.23	1	0.391	-10.86	1.66
	3 months	6 months	-1.15	2.23	1	>0.999	-7.41	5.10
	3 months	1 year	-5.76	2.28	1	0.116	-12.16	0.65
	6 months	1 year	-4.60	2.23	1	0.391	-10.86	1.66

Results of Bonferroni multiple comparisons.

**Figure 3.**
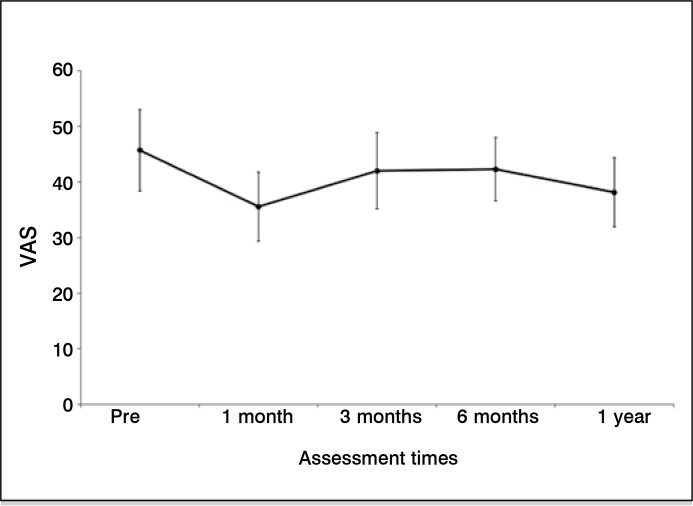
Average VAS profile and standard errors in patients with hemophilia.

**Figure 4.**
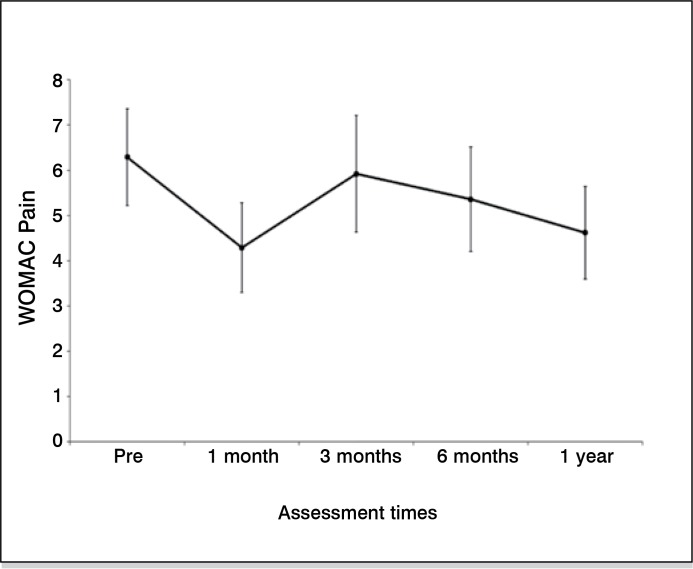
Average WOMAC Pain score and standard errors in patients with hemophilia.

**Figure 5.**
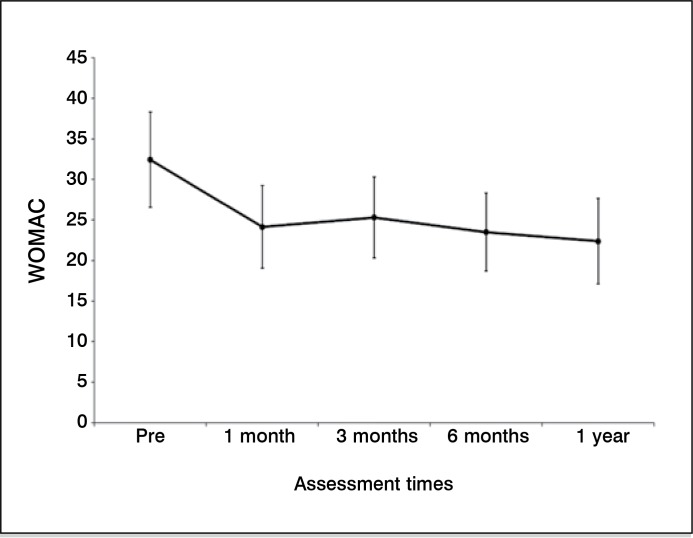
Average WOMAC score and standard errors in patients with hemophilia.

**Figure 6.**
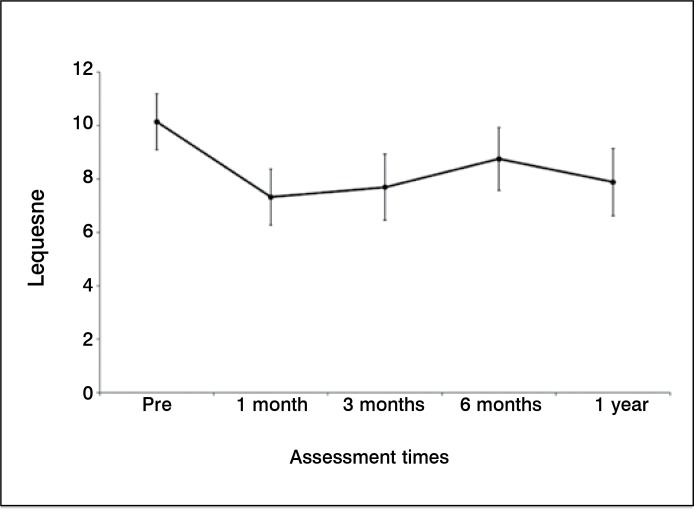
Average Lequesne score and standard errors in patients with hemophilia.

**Figure 7.**
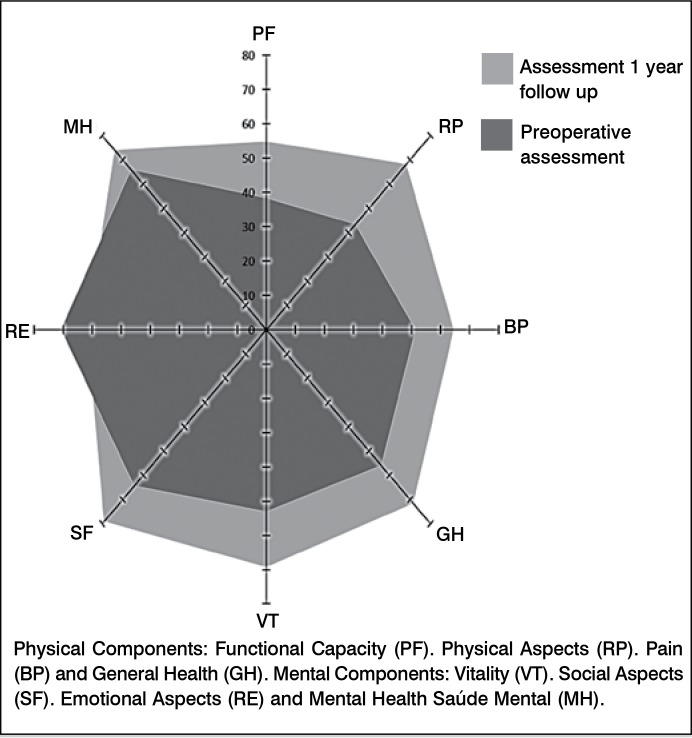
Assessment of subscales of SF-36 Quality of Life.

In Figures 3-7 we can see the group profile for pain (VAS and WOMAC pain), function (WOMAC, Lequesne) and quality of life with the physical and mental components.

## DISCUSSION

In Brazil there is a large sample of young patient with severe hemophiliac arthropathy without treatment. The Hematology Service at HC-FM-USP tracks about 200 hemophilia patients, 50% of which have orthopedic conditions. A screening of the 41 most severe cases, of which only 26 performed the exams, revealed 122 affected joints (4.7 joints per patient) of Arnold and Hilgartner grades III to V.^9^ In 39 knees (26 patients), 11 required revision implants to the first arthroplasty. The number of patients undergoing synoviorthesis is insignificant, despite its recognized eficacy.^14^^-^ 16 These young patients have repeated bleedings, severe muscle atrophy, reduced bone density (far below normal for their age), almost no articular cartilage and intense synovitis generating pain, functional limitation and bleeding. Specifically hemophiliacs' knees show hypertrophic epiphysis. Arthroplasties must be performed with care, reducing the size of the implants, in order to avoid the limitation of range of motion (ROM) in the postoperative period. Low bone mineral density of these patients can lead to early release, despite their low physical demands.^17^ Currently, with the factor replacement, these patients have normal life expectancy. Therefore, it is important to properly assess their quality of life, looking for ways to improve it, leaving arthroplasty as a last resort, if all else fails.

Arthroscopic synovectomy, performed as a second option to the non-availability of radioisotope to synoviorthesis, improves parameters of physical and mental quality of life.^18^ This improvement obtained by arthroscopic synovectomy can be reproduced by the minor washing out procedure and infiltration with triamcinolone and hyaluronic acid,^6^ primarily because we approached with this technique patients with more than one affected joint and a single procedure can improve the function of all, and by what was observed, for over a year.

This study has its limitations: 

1-It is a case series. The small number of cases (14 patients) caused bleeding in treated or untreated joints or muscle and not in the third month lead to a loss of the algofunctional results, but these patients recovered and reached better functional results than the baseline, at 6 and 12 months after the procedure. Two patients were not well at one year. One had intense knee synovitis and has been subjected to total knee arthroplasty where an extensive synovectomy was performed with major bleeding postoperatively. The other patient with osteoarthritis grade V in both ankles also scored worse on the emotional aspect (Role-Emotional, RE). The evaluation of these two patients led to poor results in the emotional component of the SF-36. The remaining patients improved in this regard (RE) of the SF-36 even one year after the procedure. (Figure 7)

2- Another limitation is the subjective nature of the completed questionnaires. However, it is still a new study quantifying the long-term effects of washing out and infiltration of the joints with triamcinolone and hylan G-F20.

3- The synoviorthesis is undoubtedly the best option to shrink and eradicate hypertrophied synovium by hemosiderin deposits. This procedure is perhaps a good option for improving function after radionuclide synovectomy, both for mechanical reasons^19^ and for visco-induction grounds.^20^ However, the possibility of simultaneous treatment of multiple joints as outpatient is appealing, leading to a progressive improvement of function and quality of life over one year. The first operated patient, already with three years of follow-up (Arnald and Hilgarten grade V), maintains the acquired results, and did not ask for a knee arthroplasty.

## CONCLUSION

The articular lavage with saline followed by infiltration of triamcinolone and hylan G-F20 is effective in the treatment of hemophilic arthropathy, especially in improving function and quality of life, even one year after the procedure.
